# The role of FOMO and mindfulness in the relationship between mobile phone dependence and sleep quality among Chinese youth: a mediating and moderating analysis

**DOI:** 10.3389/fpsyg.2025.1632396

**Published:** 2025-07-08

**Authors:** Fen Xie, Mengyu Li

**Affiliations:** ^1^School of Journalism and Communication, Guangxi Normal University, Guangxi, China; ^2^School of Communication, Shenzhen University, Shenzhen, China

**Keywords:** phone dependence, sleep quality, FOMO, mindfulness, health communication

## Abstract

**Introduction:**

Addressing the impact of problematic mobile phone use on sleep quality has become a topic of concern for researchers, but the underlying mechanisms and effective intervention strategies remain under explored.

**Methods:**

The aim of this study was to investigate the effects of cell phone dependence on sleep quality, examining the role of FOMO (fear of missing out) as a mediator and mindfulness as a moderator. A total of 529 participants under 35 years were recruited to complete the survey. The research hypothesis was tested using Model 4 and Model 8 from the PROCESS macro in SPSS.

**Results:**

Findings revealed that mobile phone dependence directly impairs sleep quality and indirectly exacerbates sleep disturbances through FOMO. Individual mindfulness traits mitigated the direct adverse effect of phone dependence on sleep quality, negatively moderated the relationship between phone dependence and FOMO.

**Discussion:**

This study expanded the Media System Dependency Theory by introducing a dual-mechanism model of “psychological mediation + individual difference moderation”, and examined the moderating role of trait mindfulness within the Chinese cultural context. It is recommended that schools and enterprises incorporate mindfulness training into digital health curricula or daily training programs.

## 1 Introduction

The rapid development of digital technology has made smartphones increasingly important to people's lives. As digital natives, the youth have been accompanied by smartphone use throughout their development. They use smartphones to access information, maintain social relationships and get entertainment. While smartphones bring substantial convenience and social resources to their lives and learning, many problems and risks accompany these benefits. Among Chinese college students, 70% to 80% of them are dependent on smartphones (Rong, 2018).

Media system dependency theory posits that the more users rely on media resources to meet their self-needs, the more central the media become in their lives, and the greater influence media will have on users' lives (Ball-Rokeach, 1975). In the digital media environment, the richness of media content and the diversification of functions have deepened users' instrumental and spiritual dependence on media, making it easier for them to develop habitual usage patterns (Xu et al., 2012). The number of people who have progressed from dependence to pathological addiction has surpassed 25% (Guo et al., 2022).

Excessive use of smartphone has imposed detrimental effects on the lives of young adults (Jeong et al., 2020), such as reducing sleep time and decreasing sleep quality (Tereshchenko et al., 2021; Acikgoz et al., 2022; Ward et al., 2017). However, the mechanisms of influence, as well as the solutions, remain underexplored and warrant further investigation (Zhu et al., 2023).

Fear of missing out (FOMO) is a typical and common phenomenon in cell phone addiction (Brown and Kuss, 2020). A meta-analysis has revealed that cell phone users across genders and age groups exhibit FOMO triggered by social media use (Zhang et al., 2021), manifested as frequent logging into social media platforms, incessant refreshing of social feeds, and an intense desire to stay informed about others' activities and updates (Przybylski et al., 2013).

In response to the issues precipitated by digital technique, the “mindfulness” approach has been introduced by researchers as a proposed solution (Liu et al., 2023). Mindfulness, defined as the capacity to observe the present moment devoid of subjective interpretation and judgment (Kabat-Zinn, 2003), has emerged as an efficacious intervention for addressing a wide array of psychological concerns (Liu et al., 2022a). Studies have indicated that mindfulness is significantly associated with mental wellbeing (Liu et al., 2022b), demonstrating its potential to mitigate the adverse effects of social media use (Poon and Jiang, 2020). Digital tools, including smartphone apps and animations, have emerged as common methods for enhancing individual mindfulness levels (Liu et al., 2021).

Although scholars have examined extensive research on smartphone dependence, sleep quality, FOMO, and mindfulness, providing valuable insights, certain gaps in the research remain.

First, existing research has focused on the direct relationship between smartphone dependence and sleep quality, with limited integration of psychological mediating mechanisms and individual difference moderators.

Second, researchers have been conducted on Western samples, with little research on Chinese. Chinese youth experience unique social pressures (such as, academic competition and group culture), and have unique digital usage patterns (such as, high frequency use of short videos) (Luo, 2022). These may lead to differences in their physical and mental health outcomes compared to Western people (Sun, 2025).

Third, present research on mindfulness mostly centers around clinical interventions or mindfulness training programs. However, for the related intervention studies, the intervention scenarios need to be further clarified and defined. Moreover, the theoretical underpinnings require greater consolidation and development.

Based on mentioned above, the current study aims to construct a moderated mediation model to explore the relationship between smartphone dependence and sleep quality among Chinese youth, as well as the mediating role of FOMO and the moderating role of mindfulness. This study extends the media system dependence theory from “instrumental dependence” to “psychological dependence” (e.g., FOMO), and introduces “individual psychological traits” (mindfulness) as moderating variables, which provide a theoretical basis for schools and enterprises to design digital mindfulness course.

## 2 Literature review and research hypotheses

### 2.1 The relationship between phone dependence and sleep quality

Research on the potential risks of mobile phone use to mental health and quality of life is on the rise (Asad et al., 2023). Excessive mobile phone use directly disrupts the sleep process. Users are often drawn to the diverse content on their mobile phones, such as video games, online videos, and social media messages, which leads to the postponement of normal bedtime and subsequently results in insufficient sleep duration. Moreover, various stimulating content on mobile phones, such as intense game plots and captivating stories, exert a strong psychological stimulus on users. This stimulus keeps the brain in a state of prolonged excitement, making it difficult to calm down within a short period (Carter et al., 2016). When using mobile phones, the light emitted by the screen interferes with the body's physiological sleep mechanism, suppressing the secretion of melatonin and disrupting an individual's physiological activities, thus preventing the body from entering a natural sleep state (Chang et al., 2015). Despite these damages to body, young people still tend to browse their mobile phones within one hour before bedtime (Krishnan et al., 2020). Building upon these established findings, the first research hypothesis is formulated as follows:?

Hypothesis 1: Mobile phone dependence has a significant impact on sleep quality.

### 2.2 FOMO as a mediating variable

FOMO is defined as the anxiety individuals experience when people fear being absent from a situation or are unable to access information or experiences they want to know. It is mainly characterized by a strong desire to know what others are doing (Przybylski et al., 2013). In the digital era, with the escalating dependence on mobile devices, FOMO has evolved into a prevalent societal concern. Study indicates that ~66% of smartphone users report experiencing FOMO, with symptom severity peaking during late-night hours and weekends (Milyavskaya et al., 2018). Social media on the phone offer highly immersive experiences that enhance cognitive and emotional engagement, thereby predisposing users to frequent mobile device use, such as repetitive phone checking, frequent social media logins, and prolonged immersion in online gaming (Alt, 2015). As such, digital technologies not only actively fulfill users' information needs but also trigger the FOMO on more information (Beyens et al., 2016). Anxiety induced by mobile device use has been linked to adverse mental health outcomes, including increased negative emotions and perceived stress (Milyavskaya et al., 2018), as well as physical discomforts (e.g., headaches, chest pain) (Baker et al., 2016) and sleep disturbances (Caba-Machado et al., 2024), more pronounced FOMO correlates with more severe sleep problems (Zhang et al., 2021). Based on these findings, the following hypothesis was proposed:

Hypothesis 2: FOMO significantly mediates the relationship between mobile phone dependence and sleep quality.

### 2.3 The moderating role of mindfulness

Mindfulness, defined as non-judgmental conscious awareness of the present moment (Kabat-Zinn, 2003), is a practice of maintaining non-evaluative perception of ongoing experiences (Bishop et al., 2004). As a psychological resource, mindfulness is used as a spiritual force in many areas of psychotherapy, where practitioners are asked to feel and experience the present moment in a non-judgmental manner rather than with specific values (Howell et al., 2008), thereby altering neurocognitive mechanisms, treating substance addictions (Li et al., 2017) and behavioral addictions (Brandtner et al., 2022), and enhancing an individual's positive emotions (Liu et al., 2020a).

In the digital era, as individuals engagement with digital media grows increasingly intense, the impact of problematic digital media use on quality of life and psychophysical health has garnered significant attention. Researchers have begun integrating mindfulness into digital lifestyles to promote healthy media use and enhance digital wellbeing (Liu et al., 2023). Studies have demonstrated that mindfulness assists individuals in redirecting their attention from external stimuli to internal metacognition (Van Gordon et al., 2014), consequently enhancing self-awareness (Chen et al., 2022), alleviating anxiety in online settings (Hu et al., 2024), regulating negative emotions (Liu et al., 2021), mitigating sleep disorders (Rusch et al., 2019), and boosting creativity (Chen et al., 2022) as well as subjective wellbeing (Liu et al., 2019). Mindfulness-based interventions have shown efficacy in mitigating postpartum depression (Liu et al., 2022c), enhancing corporate management practices (Liu et al., 2022a), and improving patient safety outcomes (Liu et al., 2022d). Given these findings, the present study hypothesizes that mindfulness may moderate the relationship between phone dependence and sleep quality, as well as phone dependence and FOMO. We proposed the following hypothesis:

Hypothesis 3: Mindfulness moderates the relationships among mobile phone dependence, FOMO, and sleep quality.
Hypothesis 3a: Mindfulness significantly moderates the direct effect of mobile phone dependence on sleep quality.
Hypothesis 3b: Mindfulness significantly moderates the first half of the mediating pathway (i.e., the effect of mobile phone dependence on FOMO).

**Figure 1:**
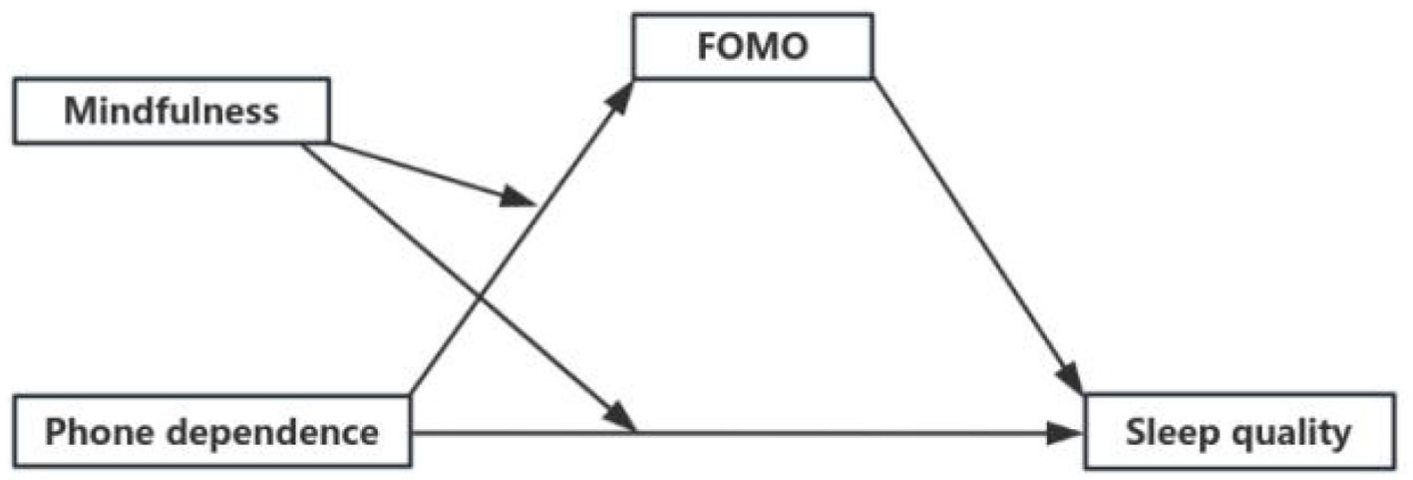
Research model.

Taken together, this study constructs a novel moderated mediation model (As shown in Figure 1), representing a core innovation of the research. The model aims to address three research questions:

First, to examine the relationship between mobile phone dependence and sleep quality;

Second, to test whether FOMO mediates the association between mobile phone dependence and sleep quality;

Third, to investigate whether mindfulness moderates both the direct and indirect relationships between mobile phone dependence and sleep quality.

## 3 Method

### 3.1 Participants

The survey was conducted following approval from the Ethics Committee of the first author's institution. Different countries adopt varying criteria to define youth (Yang and Shi, 2024). In Chinese policy, individuals aged 14 to 35 are defined as youth (Zhang, 2022). Thus, our study focuses on participants aged between 14 and 35. Before the formal survey, we carried out a pre-survey with 91 young people. The results showed that the reliability and validity of each scale were within acceptable ranges. The formal survey was conducted in a snowball fashion. In January 2025, we recruited college students to participate in the survey from WeChat groups in three universities in Guangxi, Zhejiang and Yunnan, and encouraged them to mobilize young people around them to participate in the survey. At the same time, we distributed questionnaires in university cafeterias, classrooms, as well as neighborhoods and squares in the city. The survey process was voluntary, respondents answered the questions in an informed manner, and each respondent was given a small gift after completing the survey. Data collection had lasted for one month, yielding 529 valid questionnaires. The sample comprised 30.6% male and 60.4% female participants, with 88.8% falling within the 18–25 age range. The remaining respondents were young adults below 35 years. 53.3% of participants come from rural areas, while 46.7% reported urban backgrounds.

### 3.2 Measures

#### 3.2.1 Mobile phone dependence

Mobile phone dependence was measured using the short-version Smartphone Addiction Scale (SAS-SV) (Kwon et al., 2013). The scale comprises 10 items (e.g., “I cannot complete planned tasks due to smartphone use,” “I have difficulty concentrating in class, while doing homework, or at work because of smartphone use,” “My wrist or the back of my neck hurts from smartphone use,” “I cannot tolerate being without my smartphone,” “I feel impatient or restless when my smartphone is not accessible”). Scored using a five-point Likert scale (1 = Strongly Disagree, 5 = Strongly Agree), with higher scores indicating greater dependence. In this study, Cronbach's α = 0.893.

#### 3.2.2 FOMO

FOMO Scale, originally developed by Przybylski et al. (2013), has been widely adopted in research. Li Qi adapted this scale to the Chinese cultural context (Li et al., 2019). In the present study, the localized scale revised was employed to measure participants' FOMO levels. The scale has 8 items, such as “I fear others are having more exciting experiences or gains than me,” “I worry that my friends have more rewarding experiences or achievements than I do,” “I feel upset when I find my friends are having fun without me, ” “I feel anxious when I don't know what my friends are doing” and so on. Responses were measured on a 5-point Likert scale (1 = Strongly Disagree, 5 = Strongly Agree), with higher scores reflecting greater FOMO. In this study, Cronbach's α = 0.828.

#### 3.2.3 Sleep quality

Sleep quality was measured using the Self-Rating Sleep Scale (SRSS) developed by Li (2012). Widely used in China, this scale has been demonstrated to have good reliability and validity. It consists of 10 items, such as “Do you feel that your usual sleep is sufficient?,” “Do you feel fully rested after sleep?,” “Do you doze off during the day after sleeping at night?.” Higher scores indicate poorer sleep quality. In this survey, Cronbach's α = 0.787.

#### 3.2.4 Mindfulness

Mindfulness traits of participants were measured by MAAS (Brown and Ryan, 2003). The scale comprises 15 items, such as “I often fail to consciously perceive certain emotions as they occur, only becoming aware of them after some time has passed,” “I forget a person's name almost as soon as I have been told it,” “I find it difficult to stay focused on what's happening in the present.” Responses are rated on a 5-point Likert scale (1 = almost never, 5 = almost always), with lower scores initially indicating higher mindfulness. In this study, item scores were reverse-coded so that higher total scores reflect greater mindfulness. Cronbach's α = 0.875.

### 3.3 Statistical analysis

Data were processed using SPSS and Process. First, we described the mean and standard deviation of cell phone dependence, sleep quality, mindfulness and FOMO, and explored the correlation of each variable through Pearson correlation analysis. Second, SPSS Process macros (Model 4 and Model 8) were utilized to examine the mediating role of FOMO and the moderating role of mindfulness.

### 3.4 Common method biases

Common method biases was examined using Harman's single-factor test. A principal component factor analysis without rotation was conducted on all items in this study. The results showed that the first extracted factor explained 20.341% of the total variance, which was below the 40% threshold. This indicates that common method bias is not a significant concern in this study.

## 4 Data analysis and results

### 4.1 Correlational analysis of mobile phone dependence, FOMO, sleep quality and mindfulness

Normality of the data was first tested, and all variables exhibited normal distributions, with skewness and kurtosis within acceptable ranges (skewness < |2.0|, kurtosis < |7.0|). In the collinearity diagnostics, all variance inflation factors (VIF) were below 5, indicating no severe multicollinearity. A Pearson correlation analysis was then conducted among the variables. As shown in Table 1, mobile phone dependence was significantly positively correlated with FOMO (*r* = 0.474, *p* < 0.01), sleep quality (*r* = 0.464, *p* < 0.01), and significantly negatively correlated with mindfulness (*r* = −0.087, *p* < 0.05). FOMO was significantly positively associated with sleep quality (*r* = 0.561, *p* < 0.01) and significantly negatively associated with mindfulness (*r* = −0.175, *p* < 0.01). Sleep quality was significantly negatively correlated with mindfulness (*r* = −0.198, *p* < 0.01).

**Table 1:** Correlation test of variables.

**Variables**	**M**	**SD**	**1**	**2**	**3**
Mobile phone dependence	3.42	0.77			
FOMO	3.03	0.73	0.474^**^		
Sleep quality	2.38	0.61	0.464^**^	0.561^**^	
Mindfulness	2.98	0.59	−0.087^*^	−0.175^**^	−0.198^**^

* < 0.05,

** < 0.01,

^***^< 0.001.

### 4.2 Testing the mediation effect

First, use Model 4 (a simple mediation model) from the PROCESS macro in SPSS to test the mediation effect. Gender, age, residence, and income were included as control variables in the model, as these demographic factors were considered potential confounders (Geng and Ding, 2023; Fendel et al., 2024). Table 2 presents the results of the mediation analysis. When mobile phone dependence was treated as the independent variable and sleep quality as the dependent variable, mobile phone dependence significantly predicted sleep quality in the positive direction (β = 0.465, *t* = 11.820, *p* < 0.001), indicating that higher levels of mobile phone dependence were associated with poorer sleep quality. The model explained 22.1% of the variance in sleep quality (*R*^2^ = 0.221, *F* = 29.609, *p* < 0.001). Hypothesis 1 was confirmed. When FOMO was set as the dependent variable and mobile phone dependence as the independent variable, mobile phone dependence significantly predicted FOMO in the positive direction (β = 0.486, *t* = 12.599, *p* < 0.001), indicating that higher levels of mobile phone dependence were associated with greater FOMO. This model explained 24.9% of the variance in FOMO (*R*^2^ = 0.249, *F* = 34.741, *p* < 0.001). When sleep quality was set as the dependent variable and both FOMO and mobile phone dependence as independent variables, FOMO significantly predicted sleep quality in the positive direction (β = 0.455, *t* = 11.385, *p* < 0.001), and mobile phone dependence also had a significant positive effect on sleep quality (β = 0.244, *t* = 6.062, *p* < 0.001). The model explained 37.6% of the variance in sleep quality (*R*^2^ = 0.376, *F* = 52.347, *p* < 0.001).

**Table 2:** Testing the mediation effect (*N* = 529).

**Regression equation**		**Fit indicators**			**Coefficient significance**	
**Outcome variable**	**Predictor variable**	***R***	***R*** ^2^	***F***	β	***T***
Sleep quality		0.470	0.221	29.609^***^		
	Gender				−0.036	−0.891
	Age				0.064	1.490
	Residence				0.008	0.193
	Income				−0.044	−0.995
	Mobile phone dependence				0.465	11.820^***^
FOMO		0.499	0.249	34.741^***^		
	Gender				−0.117	−2.916^**^
	Age				−0.107	−2.523^**^
	Residence				−0.021	−0.533
	Income				−0.051	−1.191
	Mobile phone dependence				0.486	12.599^***^
Sleep quality		0.613	0.376	52.347^***^		
	Gender				0.017	0.454
	Age				0.113	2.901^**^
	Residence				0.018	0.480
	Income				−0.020	−0.517
	Mobile phone dependence				0.244	6.062^***^
	FOMO				0.455	11.385^***^

* *P* < 0.05,

** *P* < 0.01,

*** *P* < 0.001.

We further examined the mediating role of FOMO in the relationship between mobile phone dependence and sleep quality using the Bootstrap method. The results are presented in Table 3. The total effect was 0.465 (BootSE = 0.039, 95% CI = [0.329, 0.428], excluding zero), indicating a significant overall impact of the independent variable on the dependent variable. The direct effect was 0.244 (BootSE = 0.048, 95% CI = [0.365, 0.565], excluding zero), demonstrating a significant direct influence of the independent variable on the dependent variable, accounting for 52.45% of the total effect. The indirect effect was 0.221 (BootSE = 0.033, 95% CI = [0.160, 0.288], excluding zero), indicating a significant mediating pathway that accounted for 47.55% of the total effect. These findings suggest that the mediator played a substantial role in this relationship.

**Table 3:** Bootstrap-based mediation effect test results.

**Effect decomposition**	**Effect**	**BootSE**	**BootLLCI**	**BootULCI**	**Proportion**
Direct effect	0.244	0.048	0.365	0.565	52.45%
Indirect effect	0.221	0.033	0.160	0.288	47.55%
Total effect	0.465	0.039	0.329	0.428	

A comprehensive analysis of the two mediational effect testing approaches described above confirms that the FOMO significantly partially mediates the relationship between mobile phone dependence and sleep quality. Hypothesis 2 was confirmed.

### 4.3 Testing the moderated mediation effects

We employed Model 8 of the PROCESS macro to examine the moderating roles of mindfulness in the relationships between mobile phone dependence and sleep quality, as well as between mobile phone dependence and FOMO. First, we standardized the variables of mindfulness, mobile phone dependence, FOMO, and sleep quality, and then calculated the interaction terms between the standardized independent variables and the moderator.

In Table 4, the regression equation for FOMO revealed that mobile phone dependence significantly predicted FOMO positively (β = 0.458, *t* = 12.017, *p* < 0.001), indicating that higher levels of mobile phone dependence were associated with greater FOMO. Mindfulness significantly predicted FOMO negatively (β = −0.135, *t* = −3.625, *p* < 0.01), suggesting that higher mindfulness was linked to lower FOMO. The interaction term between mobile phone dependence and mindfulness (β = −0.126, *t* = −3.702, *p* < 0.001) also exhibited a significant negative effect on FOMO, demonstrating that mindfulness moderated the relationship between mobile phone dependence and FOMO (*R*^2^ = 0.285, *F* = 29.724, *p* < 0.001).

**Table 4:** Moderated mediation effects (*N* = 529).

**Regression equation**		**Fit indicators**			**Coefficient significance**	
**Outcome variable**	**Predictor**	***R***	***R*** ^2^	***F***	β	***t***
FOMO		0.534	0.285	29.724^***^		
	Gender				−0.103	−2.617^**^
	Age				−0.120	−2.882^**^
	Residence				−0.035	−0.888
	Income				−0.036	−0.855
	Mobile phone dependence				0.458	12.017^***^
	Mindfulness				−0.135	−3.625^***^
	Mobile phone dependence^*^mindfulness				−0.126	−3.702^***^
Sleep quality		0.626	0.392	41.870^***^		
	Gender				0.022	0.594
	Age				0.102	2.625
	Residence				0.009	0.242
	Income				−0.012	−0.312
	Mobile phone dependence				0.241	6.056^***^
	FOMO				0.422	10.426^***^
	Mindfulness				−0.103	−2.965^**^
	Mobile phone dependence^*^mindfulness				−0.075	−2.367^*^

* *P* < 0.05,

** *P* < 0.01,

*** *P* < 0.001.

The regression equation for sleep quality showed that mobile phone dependence significantly predicted sleep quality positively (β = 0.241, *t* = 6.056, *p* < 0.001), indicating that higher mobile phone dependence was associated with worse sleep quality. FOMO significantly predicted sleep quality positively (β = 0.422, *t* = 10.426, *p* < 0.001), suggesting that greater FOMO was linked to worse sleep quality.

Mindfulness significantly predicted better sleep quality negatively (β = −0.103, *t* = −2.965, *p* < 0.01), indicating that higher mindfulness was associated with better sleep quality. The interaction term between mobile phone dependence and mindfulness (β = −0.075, *t* = −2.367, *p* < 0.05) also exhibited a significant negative effect on sleep quality, demonstrating that mindfulness moderated the relationship between mobile phone dependence and sleep quality (*R*^2^ = 0.392, *F* = 41.870, *p* < 0.001).

We further analyzed the moderating effect of mindfulness using the Bootstrap method, and the results are presented in Table 5. When mindfulness was at a low level (M−1SD), the mediating effect was 0.246 (BootSE = 0.035, 95% CI = [0.181, 0.318]), with the confidence interval excluding zero, indicating a significant mediating effect. When mindfulness was at a high level (M+1SD), the mediating effect decreased to 0.140 (BootSE = 0.036, 95% CI = [0.077, 0.216]), which remained significant. The moderated mediation effect index was −0.053 (BootSE = 0.019, 95% CI = [−0.088,−0.015]), and the confidence interval excluded zero, suggesting that mindfulness significantly and negatively moderated the mediating pathway. In summary, as mindfulness levels increase, the mediating effect gradually weakens, indicating that mindfulness plays a critical buffering role in this mediating mechanism. Hypothesis 3 was confirmed.

**Table 5:** Mediating effect of FOMO across different mindfulness levels.

**Mindfulness**	**Effect**	**BootSE**	**BootLLCI**	**BootULCI**
M-1SD	0.246	0.035	0.181	0.318
M	0.193	0.030	0.139	0.256
M+SD	0.140	0.036	0.077	0.216
Index	−0.053	0.019	−0.088	−0.015

**Figure 2:**
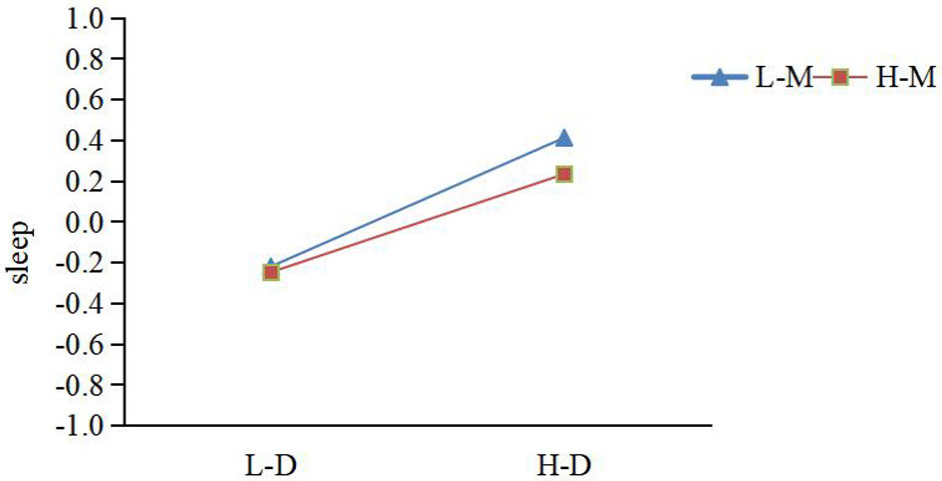
Simple slope analysis of mobile phone dependence on sleep quality across mindfulness levels (L-D = Low-Dependence; H-D = High-Dependence; L-M = Low-Mindfulness; H-M = High-Mindfulness).

To further examine the moderating role of mindfulness in the relationship between mobile phone dependence and sleep quality, we conducted simple slope tests (Aiken et al., 1991). Mindfulness and sleep quality were each categorized into high and low groups based on a criterion of plus or minus one standard deviation from the mean. The data (see Table 6 and Figure 2) revealed that when mindfulness was at a low level (M-1SD), mobile phone dependence had a significant positive effect on sleep quality (simple slope = 0.316, *t* = 6.295, *p* < 0.001). At a high mindfulness level (M+1SD), mobile phone dependence still exhibited a significant positive effect on sleep quality (simple slope = 0.166, *t* = 3.209, *p* < 0.01), though this effect was weaker. A comparison of the two groups showed that the positive impact of mobile phone dependence on sleep quality gradually diminished as mindfulness levels increased. Hypothesis 3a was confirmed.

**Table 6:** Effects of mobile phone dependence on sleep quality at different mindfulness levels.

**Mindfulness**	**Effect**	***Se***	***T***	***p***	**LLCI**	**ULCI**
M-1SD	0.316	0.050	6.295	0.000	0.217	0.415
M	0.241	0.040	6.056	0.000	0.163	0.319
M+SD	0.166	0.052	3.209	0.001	0.064	0.267

**Figure 3:**
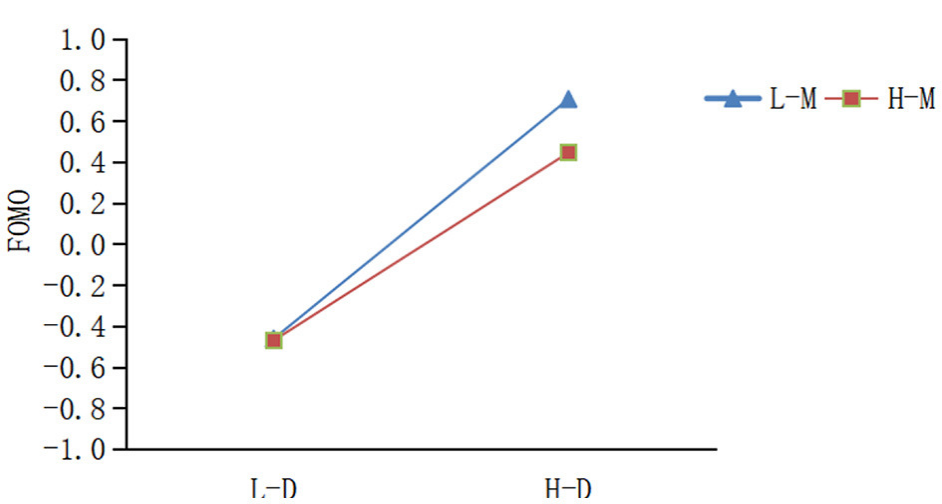
Simple slope analysis of mobile phone dependence on FOMO across mindfulness levels (L-D = Low-Dependence; H-D = High-Dependence; L-M = Low-Mindfulness; H-M = High-Mindfulness).

To further examine the moderating role of mindfulness in the relationship between mobile phone dependence and FOMO, we employed simple slope analysis. As demonstrated in Table 7 and Figure 3, when mindfulness was at a lower level (M-1SD), mobile phone dependence exerted a significant positive effect on FOMO (simple slope = 0.584, *t* = 12.165, *p* < 0.001). At higher mindfulness levels (M+1SD), mobile phone dependence still significantly predicted FOMO (simple slope = 0.332, *t* = 6.156, *p* < 0.001), the magnitude of this effect was reduced. A comparative analysis of these two groups revealed a gradual decrease in the positive association between mobile phone dependence and FOMO as mindfulness levels increased. Hypothesis 3b was confirmed.

**Table 7:** Simple slope analysis of mobile phone dependence on FOMO across mindfulness levels.

**Mindfulness**	**Effect**	***Se***	***T***	***p***	**LLCI**	**ULCI**
M-1SD	0.584	0.048	12.165	0.000	0.490	0.678
M	0.458	0.038	12.017	0.000	0.383	0.533
M+SD	0.332	0.054	6.156	0.000	0.226	0.438

## 5 Discussion

At a time when the mobile Internet is booming, mobile phones are deeply embedded in the daily life of youth groups, bringing great convenience to their work, life and study, but also accompanied by a series of negative impacts, and how to deal with the negative problems caused by phone use has become the focus of attention for many researchers.

### 5.1 Relationship between mobile phone dependence and sleep quality

Our study further confirms that mobile phone dependence negatively affects sleep quality in the youth, with the more severe the phone dependence, the worse the sleep quality. This is in line with the findings of several meta-analyses of samples from different countries, which found that the degree of cell phone dependence was correlated with sleep quality, and excessive use reduced sleep quality (Gull and Sravani, 2024; Li et al., 2020). Excessive use of phones has become a common problem among young people. In addition to prolonged use of smartphones during the day for study or work, young people also habitually use their cell phones as a means of recreation, personal space, and emotional comfort before going to bed, which usually leads to shorter sleep and increased sleep dependence (Tao et al., 2024). In the current age of digital natives, mobile phone dependence may be the new test of sleep hygiene habits (Carter et al., 2016). Although mobile devices provide convenient digital lifestyles, their long-term interference with sleep cycles leads to chronic sleep disorders, which can cause significant harm to users' physical and mental health. This necessitates that when formulating sleep management strategies, mobile phone use should be regarded as a critical indicator for optimizing sleep environments. By employing more scientific methods to mitigate its negative effects, we can reconstruct healthy sleep-wake rhythms in the digital age.

### 5.2 The mediating role of FOMO

Numerous studies have been conducted to confirm the multifaceted process by which cell phone dependence affects sleep quality, such as Tao found that smartphone dependence affects sleep quality through negative emotions and health behaviors (Tao et al., 2024), and Yang's suggestion that cell phone dependence affects sleep quality through decreased interpersonal communication and increased negative emotions (Yang et al., 2023). Other researchers argue that blue light emitted by mobile phone screens may affect melatonin levels, even low-intensity light can inhibit the release of sleep-promoting melatonin, disrupt normal circadian rhythms, and thus lead to difficulties in falling asleep (Chang et al., 2015). This study demonstrates that FOMO acts as a mediating variable in the relationship between mobile phone dependence and sleep quality, further enriching the relevant theoretical framework. FOMO on cell phone primarily stems from users' continuous attention to others' experiences or exciting information on social media; when this attention is frustrated, FOMO is likely to emerge (Przybylski et al., 2013). Researchers have explained the psychological motivations of FOMO from three theoretical perspectives: self-determination theory, belonging need theory, and flow theory (Zhao et al., 2017). According to self-determination theory (SDT), competence, autonomy, and relatedness are fundamental human psychological needs. When these needs are unmet, individuals are prone to experiencing FOMO (Przybylski et al., 2013). Belonging need theory posits that individuals have an inherent drive to integrate into groups and gain social acceptance. The proliferation of mobile internet and social media has enabled users to fulfill needs for self-expression and group affiliation (Mäntymäki and Islam, 2016), however, FOMO arises when individuals worry about failing to stay abreast of group information or being excluded from social circles (Lai et al., 2016). From the perspective of flow theory, the immersive experiences created by digital media exacerbate users' dependency on and engagement with smart devices. Users consequently develop FOMO when fearing the loss of opportunities for interaction with others or access to engaging content (Zhao et al., 2017). Young adults, in their growth and exploration phase, often face pressures from academics and career development. Their needs for competence, autonomy, and relatedness are particularly strong. However, whether these needs are met is highly uncertain. If they fail to gain satisfaction in real life, they may turn to the internet for affirmation and recognition (Mäntymäki and Islam, 2016). Meanwhile, they are accustomed to maintaining and expanding social relationships on social platforms. This high-intensity relational interaction tends to make them anxious about missing information such as others' displayed success stories, entertainment activities, thereby prompting frequent smartphone checking in an attempt to gain satisfaction and a sense of belonging (Grieve et al., 2013). The diverse content and convenient interactive features of mobile social media also captivate young adults' interest. For example, entertaining short videos may lead to prolonged smartphone use. Despite awareness of potential negative impacts, young people still spend substantial time on short-video apps and similar platforms (Zhao and Kou, 2024). These factors subtly increase psychological stress and anxiety levels, interfering with individuals' ability to relax and fall asleep, thereby compromising sleep quality (Li et al., 2020).

### 5.3 The moderating role of mindfulness

Scholars have proposed various solutions to address the negative impact of problematic mobile phone use on sleep quality. These include enhancing psychological resilience (Xie et al., 2023), strengthening stop-control (Chen and Huang, 2018), and increasing physical exercise (Peng et al., 2025). The current study showed that trait mindfulness plays a significant moderating role in the relationship between cell phone dependence, FOMO, and sleep quality. The negative effects of cell phone dependence on sleep quality and FOMO were attenuated with higher levels of mindfulness, suggesting that youths with high levels of mindfulness are more likely to detect the impulse to check their cell phones triggered by FOMO, and are better able to regulate such behaviors. At the same time, they are also able to manage cell phone dependence more effectively, which in turn improved sleep quality. These findings not only provide a theoretical basis for mindfulness training interventions in sleep contexts but also expand the media system dependency theory by introducing a dual-mechanism model of “psychological mediation+individual difference moderation,” and echoes the mindfulness-based stress reduction theory. The mindfulness-based stress reduction theory offers a theoretical framework for the relationship between mindfulness and health outcomes. This theory posits that mindfulness reduces perceived stress and emotional reactivity to stressors, with these stress-alleviating effects explaining how mindfulness influences physical and mental health (Voss et al., 2020). This further supports the logical chain of “mindfulness improves sleep by reducing FOMO” in this study.

Mindfulness exists in two forms: trait mindfulness and state mindfulness (Grundy et al., 2018), with researchers developing relevant scales as measurement tools (Chiou et al., 2022). However, scholars have paid less attention to the relationship between trait mindfulness and problematic mobile phone use, focusing mostly on state mindfulness. This study serves as a supplement to this area. Trait mindfulness refers to an individual's level of mindfulness demonstrated in daily life, which represents an individual difference and can be regarded as a personality trait. State mindfulness refers to an individual's level of mindfulness exhibited at a specific moment or during a practice session. It represents a temporary and dynamic state that can be enhanced in the short term through mindfulness practices such as meditation. Unlike trait mindfulness, state mindfulness focuses more on present-moment experiences rather than serving as a stable personal trait (Hülsheger et al., 2018). Through mindfulness training, individuals enhance their metacognitive awareness, enabling them to more promptly detect and resist addictive impulses by breaking them down into sensory, emotional, and cognitive components rather than reacting automatically in habitual ways (Garland and Howard, 2018). This study confirms that, like state mindfulness, trait mindfulness can serve as a means to address problematic social media use, thereby helping mobile phone users engage with the internet more purposefully (Fendel et al., 2024).

Notably, most mindfulness research in communication originates from Western samples, and it has not become a prominent field in China, with related studies remaining scarce. In China, there is an inherent connection between mindfulness and the concept of self-cultivation in Chinese culture. Focusing on the present world and living in the moment represent important spiritual characteristics of Chinese culture. Confucianism emphasizes concern for the present life and values real-world experiences; Taoism also advocates acceptance and attention to the present, as Lao Tzu proposed returning to and living in the moment; Buddhism places particular emphasis on maintaining awareness (Xie, 2021). The current study demonstrated that the positive effects of mindfulness traits on healthy living in the Chinese cultural context.

There are various ways in which young people can raise their level of mindfulness in their daily lives (Vargas-Nieto et al., 2024; Liu et al., 2023). Formal meditation (e.g., sitting meditation, body scans) (Liu et al., 2020b) and informal mindfulness during daily activities (e.g., eating, walking) are approaches to reduce anxiety, alleviates dependence and other addiction-related symptoms (Fendel et al., 2024). As digital technology advances, mindfulness-related apps and wearables are emerging. They can offer personalized mindfulness guidance based on situations (Iyus et al., 2025; Liu et al., 2022e), and visual/symbolic applications are more likely to attract the user's attention (Chen et al., 2023). Technology companies may consider embedding “mindfulness-friendly” features in mobile systems and applications, such as adding usage duration alerts on social platforms or inserting “content immersion reminders” on short-video platforms, to guide healthy digital use from the technological source. Schools can integrate digital mindfulness into mental health curricula, offering “mindfulness and digital literacy” workshops that combine theoretical explanations, meditation training, and APP practice to enhance students' self-regulation skills. Enterprises can incorporate it into employee health management programs, making mindfulness meditation a component of staff training. These strategies can be carried out by combining the philosophical wisdom and art forms of Chinese culture with modern mindfulness training techniques, so that such measures are in line with the cultural psychology of Chinese youth, while helping them to develop awareness, concentration and emotional management skills in their fast-paced lives.

## 6 Conclusion

This study constructed a moderated mediation model to investigate the relationships among phone dependence, FOMO, mindfulness, and sleep quality. Results revealed that phone dependence directly impairs sleep quality and indirectly disrupts sleep through FOMO. Mindfulness alleviates mobile phone dependence's direct negative effect on sleep quality. Also, it can reduce the indirect effect by negatively regulating the link between mobile phone dependence and FOMO. These findings enrich the theoretical understanding of digital behavior and mental health while providing practical guidance for addressing problematic smartphone use and sleep issues.

## 7 Limitations and future research

This study has limitations. First, this study used school students as the primary respondents (undergraduate, master's, and doctoral students) and did not adequately consider the professional youth groups, which is an oversight in our research design. Second, the data were derived from self-reports and lacked behavioral indicators and experimental data, and it is possible that self-reported data do not truly reflect the true situation of the subjects. Third, we took cross-sectional data, it can't examine the long-term links among mobile phone dependence, FOMO, mindfulness, and sleep quality. Fourth, the findings are mainly applicable within Chinese society, but cannot explain the situation in other countries or regions.

Future research can improve in the following ways. First, clearly distinguish between students and working youth in surveys, considering their unique characteristics. Second, we should adopt a more comprehensive approach to data collection, such as integrating users' internet behavioral data for analysis, and conducting intervention experiments to obtain more accurate conclusions. Third, use fixed samples for long-term studies to track the relationships between smartphone use, sleep and other issues, and explore their long-term impacts on youth psychological and physical health. Fourth, conduct comparative analyses of mobile phone dependence and its impacts on youth across different regions and cultural contexts.

## Data Availability

The raw data supporting the conclusions of this article will be made available by the authors, without undue reservation.
